# An automated microscopy workflow to study *Shigella*–neutrophil interactions and antibiotic efficacy *in vivo*

**DOI:** 10.1242/dmm.049908

**Published:** 2023-05-10

**Authors:** Arthur Lensen, Margarida C. Gomes, Ana Teresa López-Jiménez, Serge Mostowy

**Affiliations:** ^1^ Department of Infection Biology, London School of Hygiene and Tropical Medicine, London WC1E 7HT, United Kingdom; ^2^ Département de Biologie, École Normale Supérieure, PSL Université Paris, F-75005, Paris, France

**Keywords:** Antibiotics, High-content microscopy, Neutrophils, *Shigella*, Zebrafish

## Abstract

*Shigella* are Gram-negative bacterial pathogens responsible for bacillary dysentery (also called shigellosis). The absence of a licensed vaccine and widespread emergence of antibiotic resistance has led the World Health Organisation (WHO) to highlight *Shigella* as a priority pathogen requiring urgent attention. Several infection models have been useful to explore the *Shigella* infection process; yet, we still lack information regarding events taking place *in vivo*. Here, using a *Shigella*-zebrafish infection model and high-content microscopy, we developed an automated microscopy workflow to non-invasively study fluorescently labelled bacteria and neutrophils *in vivo*. We applied our workflow to antibiotic-treated zebrafish, and demonstrate that antibiotics reduce bacterial burden and not neutrophil recruitment to the hindbrain ventricle. We discovered that nalidixic acid (a bactericidal antibiotic) can work with leukocytes in an additive manner to control *Shigella flexneri* infection and can also restrict dissemination of *Shigella sonnei* from the hindbrain ventricle. We envision that our automated microscopy workflow, applied here to study the interactions between *Shigella* and neutrophils as well as antibiotic efficacy in zebrafish, can be useful to innovate treatments for infection control in humans.

## Teaser

**Summary:** Development of an automated image analysis workflow to enable fast and reliant immune cell counting in *Shigella*-infected zebrafish larvae, and to reveal the *in vivo* impact of antibiotics on *Shigella*–neutrophil interactions.

## INTRODUCTION

*Shigella* species are the causative agent of shigellosis (also known as bacillary dysentery) and responsible for ∼200,000 deaths per year (Khalil et al., 2018; Kotloff et al., 2013, 2018). Among *Shigella* species, *Shigella flexneri* and *Shigella sonnei* are the most prevalent infecting humans. While *S. flexneri* mostly impacts low to middle income countries (LMICs), *S. sonnei* is more common in rich and industrialized countries (Livio et al., 2014; Torraca et al., 2020). No effective vaccine is currently available to prevent shigellosis (Mani et al., 2016) and, thus, antibiotics are the most efficient treatment to avoid severe disease. However, the emergence of antibiotic resistance is raising significant concerns and led the World Health Organisation (WHO) to highlight *Shigella* as a priority pathogen requiring urgent attention (World Health Organization, 2017).

Considering that zebrafish larvae have no adaptive immune system, the zebrafish infection model is highly suited for studying how innate immune cells respond to *Shigella* infection (Duggan and Mostowy, 2018; Gomes and Mostowy, 2020; Mostowy et al., 2013; Torraca and Mostowy, 2018). Neutrophils are crucial to control *Shigella in vivo* (Mostowy et al., 2013; Raqib et al., 2002)*,* but their population declines during infection due to the release of neutrophil extracellular traps (NETs) and their overall exhaustion, a condition called neutropenia. In the case of zebrafish infection with a non-lethal dose of *Shigella*, the neutrophil population can be replenished ∼48 h post infection (hpi) by emergency granulopoiesis (Gomes et al., 2022 preprint; Willis et al., 2018). In the case of human infection, we need to understand how antibiotics may impact *Shigella*–neutrophil interactions *in vivo* to develop better therapeutic strategies.

In this study, we exploited the *Shigella*-zebrafish infection model and the imaging power of high-content microscopy to investigate how antibiotics work in combination with neutrophils to combat bacterial infection *in vivo*. We first developed a workflow to automate image analysis capturing bacterial burden and neutrophil dynamics in zebrafish over time. Using our automated microscopy workflow, we tested four different antibiotics on *Shigella*-infected zebrafish and showed how they impact on bacterial burden, neutrophil recruitment and bacterial dissemination from the hindbrain ventricle (HBV). These results highlight the importance of testing antibiotic efficacy *in vivo,* and suggest a powerful approach to perform high-throughput drug screening on infected zebrafish to innovate treatments for infection control in humans.

## RESULTS

### Non-invasive characterization of bacterial burden *in vivo* over time

**Fig. 1.**
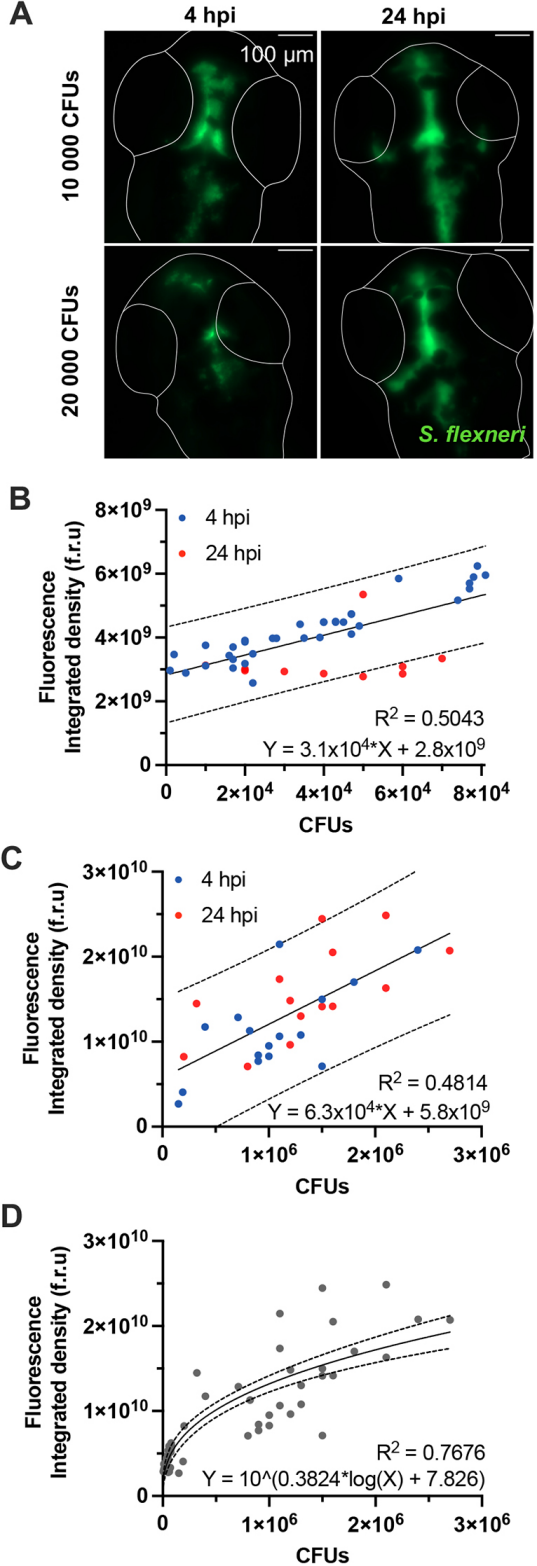
**Quantification of bacterial burden in the HBVs.** Data presented here were collected in the HBVs of *S. flexneri* M90T-infected 2 dpf zebrafish larvae. (A) Representative images of infected larvae injected with 10,000 or 20,000 CFUs at 4 or 24 hpi. Images are sum slice *z*-projections. Scale bars: 100 µm. *S. flexneri* M90T is shown in green. Outlines of larvae HBVs are shown in white. Plotted in B-D is the total fluorescence in the *z*-stack image of *S. flexneri* M90T-infected zebrafish larvae HBVs, at different infectious doses, measured at 4 (blue dots) or 24 hpi (red dots), and correlated to the precise number of CFUs (experimentally assessed). (B) Correlation between total fluorescence and precise number of CFUs in larvae with low bacterial burden (*n*=45). (C) Correlation between total fluorescence and precise number of CFUs in larvae with high bacterial burden (*n*=45). (D) Correlation between total fluorescence and precise number of CFUs in larvae with low or high bacterial burden (*n*=90). Solid lines indicate linear (B,C) or log (D) regressions. Dashed lines indicate 95% confidence intervals.

To quantify bacterial burden from infected zebrafish, larvae are usually dissociated in 0.1% Triton X-100, serially diluted and spread on a tryptic soy agar (TSA) plate, in order to later count colony-forming units (CFUs). However, this method is invasive and cannot be used to analyse the same larva over time. To non-invasively quantify bacterial burden from infected larvae over time, we used high-content microscopy to image up to 96 larvae per session. Larvae were embedded upside down in low melting agarose, with the HBV against the glass bottom of a 96-well plate, to allow imaging of the entire HBV using a 250 µm *z*-stack. We designed an ImageJ macro to calculate the total fluorescence in the HBV of zebrafish infected with the GFP-expressing *S*. *flexneri* 5a strain M90T (Fig. 1A), and total fluorescence was compared to the manually quantified number of CFUs from the same larva. This automated microscopy workflow was performed on larvae at 2 days post fertilisation (dpf) injected with a low (10,000 CFUs) or high (20,000 CFUs) input of *S. flexneri* and imaged at 4 and 24 h post-infection (hpi), to provide a broad range of infection conditions and bacterial burdens. Automated analysis of total HBV fluorescence was weakly correlated to results experimentally obtained by counting CFUs, with an R^2^ of ∼0.50 for both low and high bacterial burden (Fig. 1B,C). Results obtained from larvae with a lower bacterial burden have a smaller confidence interval – and, thus, a greater accuracy – than those from larvae comprising a high bacterial burden, independently of the observed timepoint. Consistent with this, the range in which CFUs can be associated with total HBV fluorescence is narrow, suggesting that automatic quantifications in larvae with a low bacterial burden are more precise than those in larvae with a high bacterial burden (Fig. 1D). We, therefore, chose to differentiate between low and high *S. flexneri* burden on the basis of total HBV fluorescence. Although automated image analysis failed to provide precise CFU quantifications for the range of bacterial inputs tested here, particularly for very high infection doses, it can enable qualitative description of bacterial burden (e.g. to infer bacterial replication or clearance) in a non-invasive manner.

Considering limitations in quantification of bacterial burden through fluorescence intensity (which are specific to our experimental setup), we hypothesized that dark melanophores in the HBV of the developing larvae are able to partially block acquisition of fluorescence. To test this, we treated larvae with phenylthiourea (PTU) to inhibit melanisation (Karlsson et al., 2001), injected them with low or high input of *S. flexneri* for imaging at 4 and 24 hpi. In this case, automated analysis showed that the linear correlation between total HBV fluorescence is not improved in transparent PTU-treated larvae (Fig. S1). We expect that future work using higher magnification in order to enable better resolution to image HBVs will enable a more precise, quantitative description of the bacterial burden. However, the time of acquisition would also increase, thereby, limiting experimental throughput.

### Use of automated microscopy to quantify zebrafish neutrophils *in vivo*

**Fig. 2.**
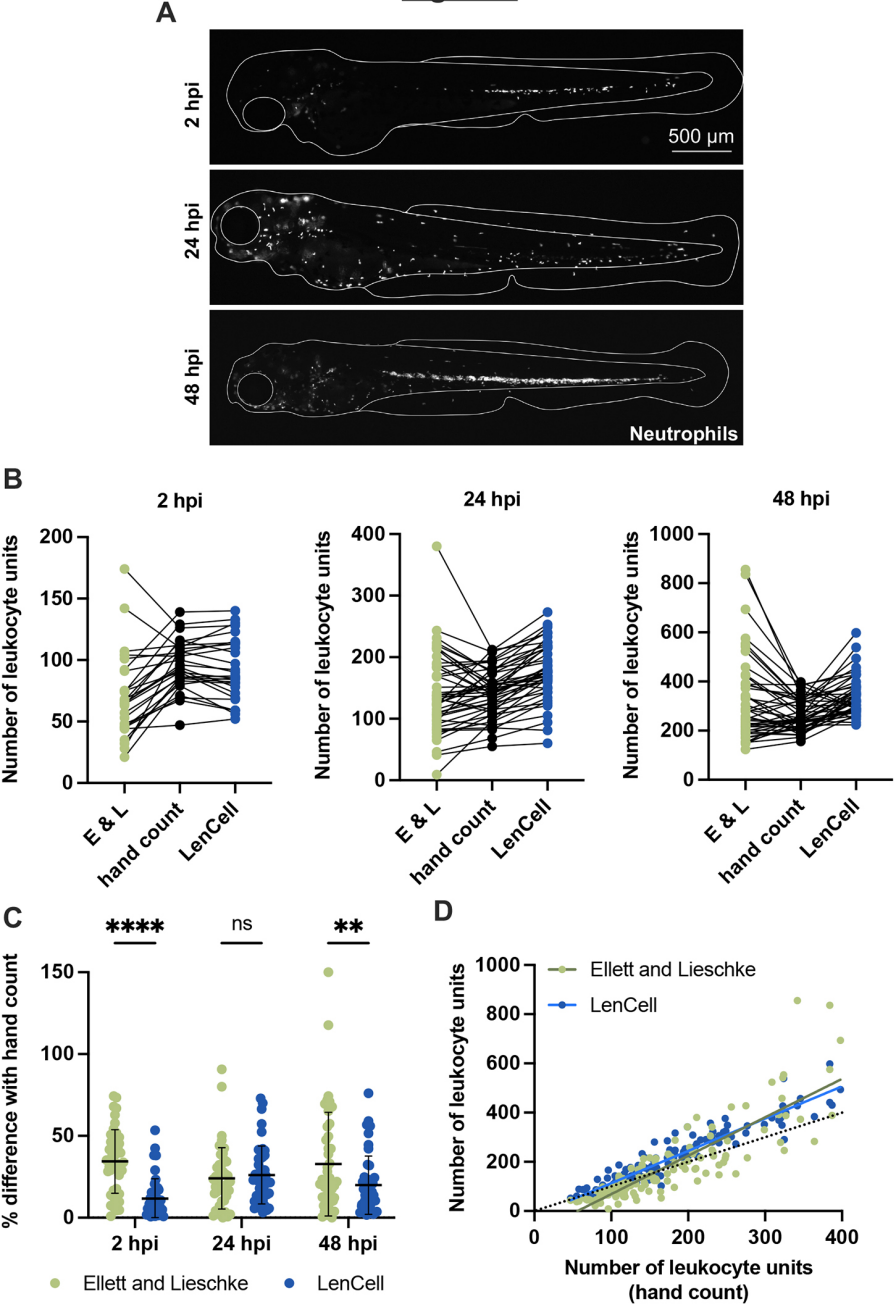
**Quantification of zebrafish neutrophils.** All data presented here were collected from *S. flexneri* M90T-infected 2 dpf zebrafish larvae (2500 CFUs) at 2, 24 and 48 hpi, or from PBS-injected larvae. (A) Representative images of infected Tg(*lyz*::DsRed)*^nz50^* larvae. Assessment of neutrophil numbers in larvae at 48 hpi during emergency granulopoiesis is particularly challenging. Neutrophils are shown in white. Scale bar: 500 µm. (B) Comparison of leukocyte units/neutrophil numbers counted by hand (black), using the Ellett and Lieschke (E&L) method (green) or the LenCell macro (blue) in infected larvae at 2, 24 and 48 hpi. Leukocyte unit quantifications are provided. Data are presented without post-processing correction; *n*=44 per method. (C) Leukocyte unit quantification in infected larvae at 2, 24 and 48 hpi. Comparison between hand count of leukocyte units/neutrophils, and that using the Ellett and Lieschke method (green) or the LenCell macro (blue). Plotted values are the differences in percentage between the count using the macro and by hand for every sample (*n*=44 per method). **P<0.01; ****P<0.0001; ns, not significant. Two-way ANOVA with Sidak's multiple comparison test. Black bars: mean±s.e.m. (D) Correlation of leucocyte units between hand count (*x*-axis), and use of the Ellet and Lieschke method count (green) and the LenCell macro (blue) (both *y*-axis); *n*=132 per method. Black dotted line indicates the identity line. Green line indicates the linear regression on the Ellett and Lieschke method results (slope=1.561). Blue line indicates the linear regression of the LenCell macro results (slope=1.312).

To characterize interactions between *S. flexneri* and zebrafish neutrophils, we infected larvae with neutrophils expressing the fluorescent protein DsRed [Tg(*lyz*::DsRed)*^nz50^*] and imaged them between 2 and 48 hpi using high-throughput microscopy. Larvae were aligned on their side and embedded in low melting agarose in a 96-well plate, to capture fluorescent neutrophils on a single *z*-plane. Although imaging of neutrophils is relatively fast, the time required to manually analyse images (∼1.5 min per image under conditions tested by us) is a significant limitation. To rapidly detect neutrophils without user bias, automated image analysis workflows are required. A pioneering method, hereafter referred to as the ‘Ellett and Lieschke’ method, uses an area calculation and averaging workflow to return a proxy of neutrophil numbers (leukocyte units) per image after correction (Ellett and Lieschke, 2012). Although this method can be relatively slow (analysis time of ∼1.0 min per image) and error-prone (error rates in determining leukocyte units in the range of 35% to 75% under the conditions tested by us) (Fig. S2), values can be improved by applying a correction factor that requires generation of a standard curve. We observed that values are particularly unreliable when analysing images of zebrafish undergoing emergency granulopoiesis, during which neutrophils are densely packed together in the aorta–gonad-mesonephros (AGM) (Fig. 2A).

To quantify zebrafish neutrophils rapidly and precisely from large datasets, we developed an ImageJ macro called ‘LenCell’. LenCell automatically applies a band-pass filter to remove high and low background frequencies, and to detect bright spots (in this case fluorescent neutrophils) in the cleaned images. To characterize the efficiency of LenCell at whole-animal level, transgenic larvae with DsRed-neutrophils were imaged on a single plane during a non-lethal *S. flexneri* infection (2500 CFUs; Fig. 2A). We then assessed the efficiency of both the Ellett and Lieschke method (without data normalisation) and the LenCell macro (optimized by iteratively identifying image analysis parameters) by comparing leukocyte units with neutrophil numbers counted by hand (Fig. 2B)*.* By determining the mean error between both methods from 132 images of *S. flexneri-*infected larvae, we found that both the Ellett and Lieschke method and LenCell are relatively accurate (Fig. 2C). However, although differences between automated quantification methods for zebrafish undergoing neutropenia (24 hpi) was negligible, LenCell appears to be more accurate when the immune system is at homeostasis (2 hpi) and also when it undergoes emergency granulopoiesis (48 hpi; Fig. 2C). As one limitation, LenCell – like the Ellett and Lieschke method – can overestimate the number of neutrophils in larvae that present high (>250) neutrophil numbers (Fig. 2D). Moreover, the positioning of larvae for imaging is important to obtain reliable results, in particular to acquire single *z*-plane images that will capture the vast majority of neutrophils, with limited overlapping of cells. We also adapted LenCell to automatically detect leukocyte units in images of the HBV acquired by *z*-stacking by using a maximum projection of *z*-stack images. In this case, LenCell performed similar compared with hand counts, the mean error being ten leukocyte units (Fig. S2B).

Overall, these results show that both the Ellett and Lieschke method and LenCell can accurately quantify leukocytes in zebrafish larvae. LenCell is six times faster than the Ellet and Lieschke method, as it can automatically analyse ∼60 images per minute. Furthermore, LenCell allows for accurate neutrophil quantification under multiple conditions – including homeostasis or under uninfected conditions, neutropenia, emergency granulopoiesis, HBV infection – and can be used to test a wide variety of bacterial inputs.

### Testing the interplay between antibiotics, bacterial burden and neutrophil response

To investigate the interplay between antibiotics and neutrophils during *Shigella* infection, we selected four antibiotics clinically used to treat shigellosis in humans: nalidixic acid (NAL), chloramphenicol (CM), azithromycin (AZI) and trimethoprim (TPI). These antibiotics have different mechanisms of action in that NAL and TPI are bactericidal, whereas CM and AZI are bacteriostatic (https://go.drugbank.com). TPI, CM and AZI have been shown to affect neutrophil function in humans: TPI can trigger neutropenia (De Manzini et al., 1990), whereas CM and AZI can reduce the release of neutrophil extracellular traps (NETs) (Bystrzycka et al., 2017); CM can additionally induce oxidative stress in neutrophils (Páez et al., 2008).

Considering differences in susceptibility to antibiotics between *S. flexneri* and *S. sonnei* (CDC, 2010; EUCAST, 2022), we performed zebrafish infection experiments using both bacterial species. We hypothesized that their differences in antibiotic susceptibility trigger a range of different behaviours in antibiotic-treated zebrafish, which may provide more information on the role of antibiotic–neutrophil interactions during *Shigella* infection. We first determined the minimum inhibitory concentrations (MICs) of drugs against *S. flexneri* and *S. sonnei in vitro*, and observed that *S. sonnei* is less sensitive to antibiotics than *S. flexneri* (Table S1). Therefore, for experiments performed *in vivo*, we used concentrations of 8 µg/ml for NAL, 5 µg/ml for CM, 16 µg/ml for AZI and 4 µg/ml for TPI because they are effective against both *Shigella* species and have no effect on larval development.

**Fig. 3.**
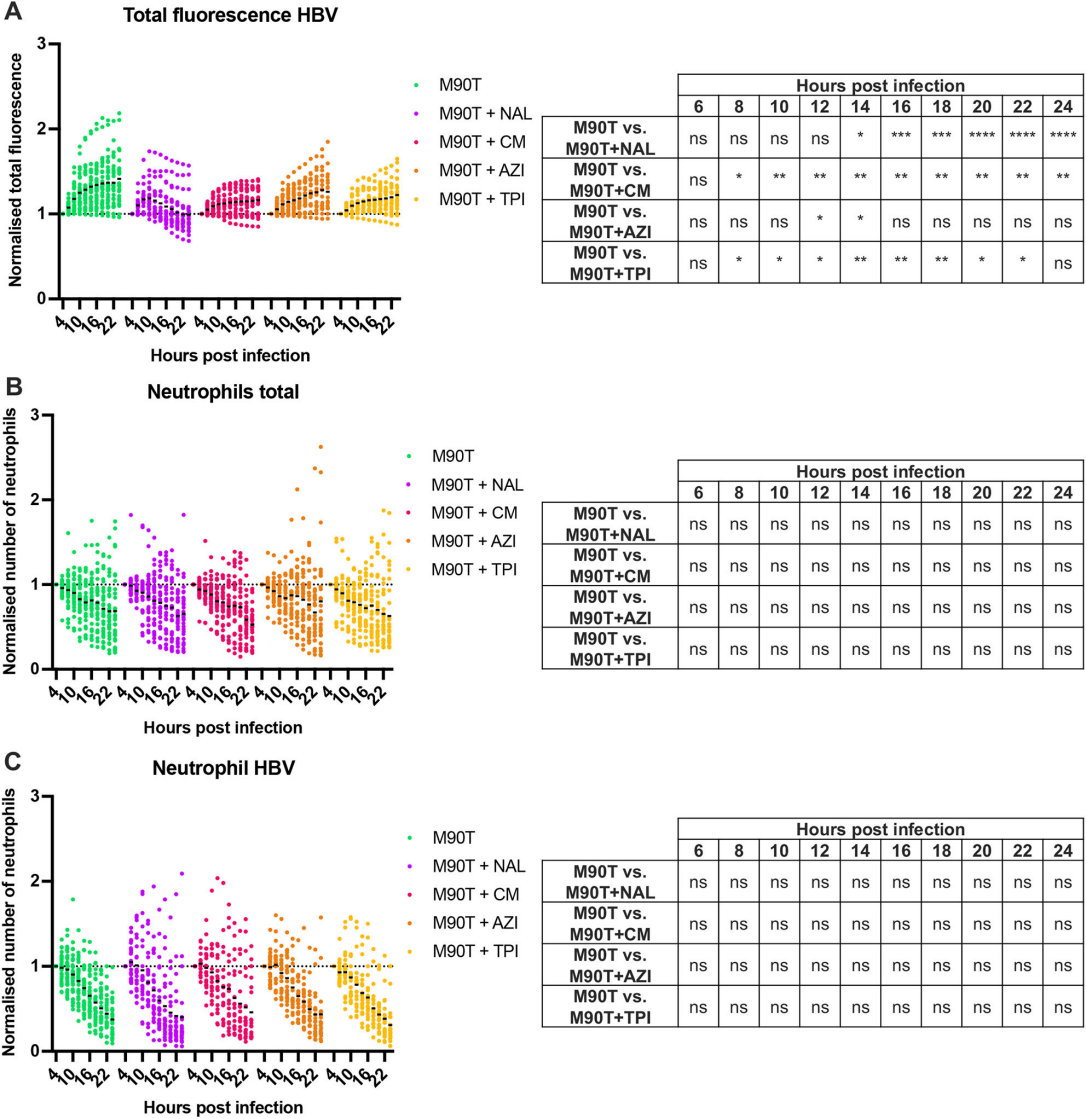
**Impact of antibiotics on bacteria and neutrophils during infection.** All data presented here were collected from *S. flexneri* M90T-infected 2 dpf zebrafish larvae that had either not been treated (green), or had been treated with nalidixic acid (NAL, purple), chloramphenicol (CM, pink), azithromycin (AZI, orange) or trimethoprim (TPI, yellow). (A) Normalised total bacterial fluorescence in HBVs. Data were normalized to the first timepoint (4 hpi) and pooled from three independent experiments using *n*>10 larvae per condition per experiment. ns, non-significant; *P<0.05; **P<0.01; ***P<0.001; ****P<0.0001. Two-way ANOVA with Dunnett's multiple comparisons test. (B) Normalised neutrophil quantification at whole larvae level. Data were normalized to the first timepoint (4 hpi) and pooled from three independent experiments using *n*>10 larvae per condition per experiment. ns, non-significant. Two-way ANOVA with Dunnett's multiple comparisons test. (C) Normalised neutrophil quantification in HBVs. Data were normalized to the first timepoint (4 hpi) and pooled from three independent experiments using *n*>10 larvae per condition per experiment. ns, non-significant. Two-way ANOVA with Dunnett's multiple comparisons test.

To decipher the interplay between antibiotics, bacterial burden and neutrophil response, infections were performed in 2 dpf zebrafish larvae with DsRed-labelled neutrophils. Larvae were injected in the HBV with a low input (10,000 CFUs) of *S. flexneri* (Fig. 3; Fig. S4A-C) or *S. sonnei* (Fig. S3; Fig. S4D-F) and imaged using high-content microscopy every 2 h for 24 hpi. As determined using LenCell, *S. flexneri* burden is significantly reduced over time in the presence of antibiotics, except for AZI (Fig. 3A; Fig. S4A). Although the concentration of antibiotics used was twice that of the *in vitro* MIC for *S. sonnei*, it was not enough to completely inhibit bacterial growth. This suggests that antibiotic concentrations used for larval immersion in our assays are insufficient to reach the MIC for bacteria within HBVs, or that infecting bacteria are less susceptible to antibiotics within HBVs. In agreement, we failed to capture a significant reduction of *S. sonnei* in the presence of antibiotics *in vivo* (Fig. S3A; Fig. S4D).

*S. flexneri* infection has been shown to reduce the neutrophil population within the whole zebrafish (Gomes et al., 2022 preprint; Willis et al., 2018). Treatment with antibiotics did not significantly impact the neutrophil population during infection with either *S. flexneri* or *S. sonnei*, i.e. neutrophil population was reduced under all conditions (Fig. 3B; Fig. S3B; Fig. S4B,E). To assess neutrophil recruitment to the HBV, LenCell was adapted to quantify cells from images of the HBV that had been acquired by *z*-stacking simultaneously with measurements of the bacterial burden. These measurements showed that antibiotic treatment did not significantly impact neutrophil recruitment to the HBV (Fig. 3C; Fig. S3C, Fig. S4B,E).

### NAL and the immune system work in an additive manner to control *S. flexneri* infection

**Fig. 4.**
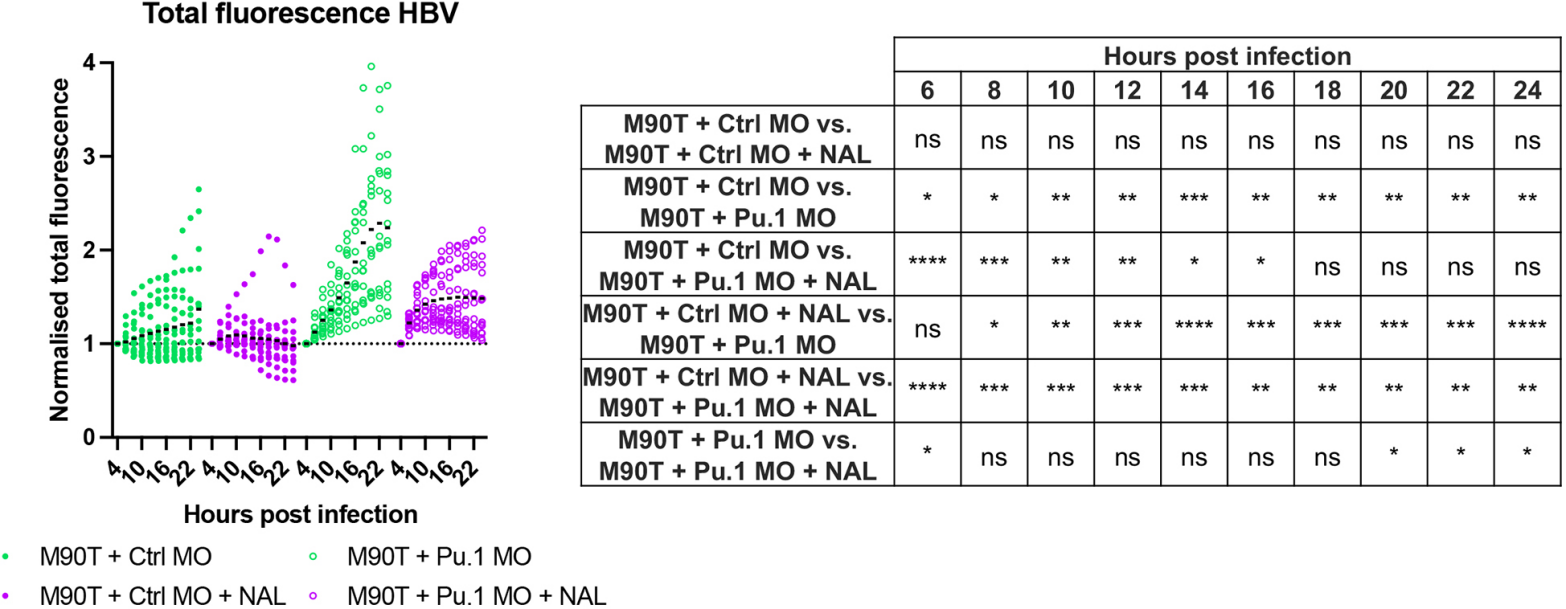
**Nalidixic acid and leukocytes work in an additive manner to control *S. flexneri* infection.** All data presented here were collected from control-injected (Ctrl MO, full circles) and *pu.1* morpholino-injected larvae (Pu.1 MO, open circles). *S. flexneri* M90T-infected 2 dpf zebrafish larvae were either not treated (green) or treated with nalidixic acid (NAL, purple). Pooled data are from three independent experiments (*n*>3 larvae per condition per experiment). ns, non-significant; *P<0.05, **P<0.01, ***P<0.001, ****P<0.0001. Two-way ANOVA with Tukey's multiple comparisons test.

Considering that NAL had a significant impact on reducing bacterial burden *in vivo*, we hypothesised that NAL and leukocytes work together to control *Shigella* infection. To test this, we performed *pu.1* morpholino (MO) injections to ablate leukocytes (Tenor et al., 2015) in larvae of the transgenic zebrafish line Tg(*lyz*::DsRed)*^nz50^* (Fig. S5A,B). Control (Ctrl) and *pu.1* MO zebrafish larvae were injected with the *S. flexneri* 5a strain M90T (Fig. 4) or the *S. sonnei* strain 53G (Fig. S5), in the presence or absence of NAL. These results demonstrated that bacteria replicate uncontrollably in the absence of both leukocytes and NAL (Fig. 4A; Fig. S5C). However, in the presence of NAL, the burden of *S. flexneri* was reduced in both control and *pu.1* MO larvae. Importantly, the reduction was not to the same level as when leukocytes are present – as demonstrated by significant differences at every timepoint analysed. Similar results were obtained after infection with *S. sonnei* 53G (Fig. S5). However, in this case, statistical differences testing for the effect of NAL on the burden of *S. sonnei* with or without leukocytes were not captured. This was expected considering our previous results showing that NAL does not significantly reduce *S. sonnei* 53G infection *in vivo* (Fig. S3). In summary, these results demonstrate that NAL and leukocytes work in an additive manner to control *S. flexneri* infection.

### NAL inhibits bacterial dissemination from the HBV

To further test the impact of antibiotics on infection control *in vivo*, we investigated the dissemination of bacteria from the HBV. In the case of zebrafish HBV infection, we define events of dissemination as when *Shigella* is able to cross the blood-brain barrier and spread in the spinal cord. These events can be easily observed when green fluorescence appears in the spinal cord of larvae infected with GFP*-Shigella*. The mechanisms underlying *Shigella* dissemination in zebrafish are mostly unknown and studying the impact of antibiotics on this process by using zebrafish may provide important clues about the control of dissemination in humans.

**Fig. 5.**
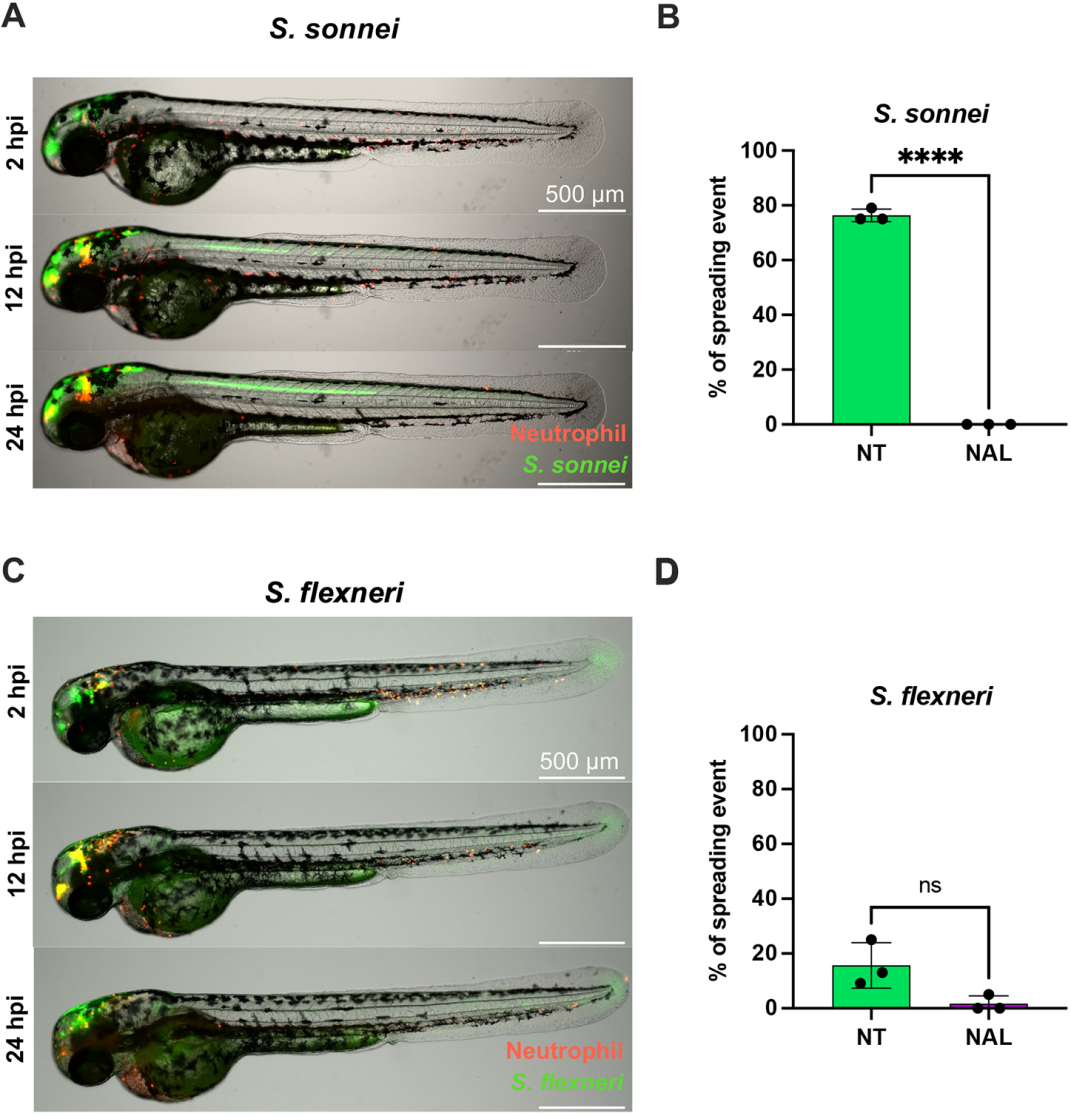
**Nalidixic acid prevents *Shigella* dissemination in zebrafish larvae.** All data presented here were collected from *S. sonnei* 53G- or *S. flexneri* M90T-infected 2 dpf zebrafish larvae that had either been not treated (NT, green) or had been treated with nalidixic acid (NAL, purple). (A) Representative spreading event in a larva infected with *S. sonnei* 53G GFP (green), with neutrophils labelled in red. The same larva was imaged at 2, 12 and 24 hpi, while being embedded in low melting agarose for the duration of the experiment. The green line through the larval spine indicates the spreading event. Scale bars: 500 µm. (B) Percentage of spreading events in larvae infected with *S. sonnei* 53G over the course of 24 hpi. Data were pooled from three independent experiments, with *n*>10 larvae per condition per experiment. ****P<0.0001. Unpaired two-tailed Student's *t*-test. (C) Representative spreading event in a larva infected with *S. flexneri* M90T GFP (green), with neutrophils labelled in red. The same larva was imaged at 2, 12 and 24 hpi, while being embedded in low melting agarose for the duration of the experiment. The green line through the larval spine indicates the spreading event. Scale bars: 500 µm. (D) Percentage of spreading events in larvae infected with *S. flexneri* M90T over the course of 24 hpi. Data were pooled from three independent experiments, with *n*>10 larvae per condition per experiment. ns, non-significant. Unpaired two-tailed Student's *t*-test.

While dissemination from the HBV in zebrafish is frequent during *S. sonnei* infection (∼75%, Fig. 5A,B), during *S. flexneri* infection it is significantly less frequent (∼15%, Fig. 5C,D). Strikingly, treatment with NAL significantly reduced the occurrence of dissemination events to 0% in *S. sonnei-*infected larvae and to ∼2% in *S. flexneri-*infected larvae (Fig. 5B,D).

## DISCUSSION

The emergence of antibiotic resistance in *Shigella* is a global concern and solutions will require development of innovative antimicrobial strategies. The use of zebrafish has previously helped to illuminate the crucial role of neutrophils in *Shigella* infection control (Duggan and Mostowy, 2018; Gomes and Mostowy, 2020; Mostowy et al., 2013; Torraca and Mostowy, 2018). In this report, we used high-content microscopy and developed an automated workflow to non-invasively measure bacterial burden and neutrophils in infected larvae over time. Our workflow significantly accelerated image analysis and rapidly increased the throughput of data. Given that our used workflow is flexible, it can be adapted to a wide variety of fluorescently labelled bacterial species – as long as the standard curve comparing total fluorescence and CFUs is generated for each species under investigation – or to zebrafish immune cells with bright enough fluorescent markers. Our attempts to use this workflow to count hematopoietic stem cells with very dim fluorescent markers – for example, with Tg(*runx1*:mCherry) – were not reliable. However, the mounting of zebrafish larvae in low melting agarose remained a rate-limiting step, as larvae needed to be oriented by hand in order to provide images compatible with our LenCell macro. However, we expect that future developments in fluidics or robotic mounting can improve the throughput of our workflow.

Our qualitative analysis of *S. flexneri* burden in zebrafish showed that antibiotic MICs determined *in vitro* are not sufficiently high to reach MICs in the HBV by immersion. This might be due to mechanisms of antibiotic storage by specific compartments of the cells (such as lipid droplets) followed by a controlled release of the drugs in the organism (Greenwood et al., 2019), or reduced penetration into the HBV. Another possibility is that infecting bacteria have phenotypic shifts – such as differences in growth or metabolism – as compared to planktonic bacteria, which could require the use of higher antibiotic concentrations *in vivo* (Certain et al., 2017; Fantin et al., 1991). Our results indicate that antibiotics are working with the immune system in an additive manner to control *S. flexneri* infection. These results after the use of antibiotics can be compared to those after the use of the P1 bacteriophage (Huan et al., 2022) or predatory bacteria (*Bdellovibrio bacteriovorus*) to treat against *S. flexneri* infection in zebrafish (Willis et al., 2016). In the latter case, injection of *Bdellovibrio* triggers neutrophil recruitment and promotes direct interaction of *Bdellovibrio* with *S. flexneri*, whereas antibiotics diluted into the bath water require time to diffuse through the larvae to reach the bacteria. It is, thus, of great interest to precisely compare the mechanism and impact of protection offered by antibiotics with those provided by *Bdellovibrio* when using our *Shigella-*zebrafish infection model.

### Concluding remarks

In the future, our automated microscopy workflow can help screen drug libraries, study clinically relevant *Shigella* strains and test the role of host genes underlying neutrophil biology. It also has the great potential to define efficacy of antibiotics *in vivo*, as well as the concentration needed to reach the MIC in the zebrafish HBV by immersion. In this way, our workflow may help to define new therapeutic strategies to combat *Shigella* infection in humans. Our workflow can be viewed as an important step towards the development of personalised medicine and may, one day, be useful to generate a database of antibiotics and their efficacies against different bacterial pathogens *in vivo*.

## MATERIALS AND METHODS

### Ethics statement

Animal experiments were performed according to the Animals (Scientific Procedures) Act 1986 and approved by the Home Office (Project license: P4E664E3C). All experiments were conducted up to 5 days post fertilisation (dpf).

### Bacterial strains and zebrafish lineages

*Shigella flexneri* M90T and *Shigella sonnei* 53G strains that both carry a GFP-expressing plasmid containing a carbenicillin resistance cassette were used (Mostowy et al., 2010; Valdivia et al., 2006). Bacteria were grown at 37°C in tryptic soy broth (TSB) or on tryptic soy agar (TSA) plates including 0.01% Congo Red to select for clones with a functional type III secretion system (T3SS) system. The growth medium was supplemented with 100 µg/ml carbenicillin.

Outbred wild-type AB zebrafish and the neutrophil transgenic zebrafish line Tg(*lyz*::DsRed)*^nz50^* (Hall et al., 2007) were used. Eggs were obtained from naturally spawning zebrafish. Fish were kept at 28.5°C in 0.5× E2 medium and exposed to 14 h of daylight per 24 h. For experiments involving phenylthiourea (PTU)-treated larvae, embryos were incubated in E2 medium+0.2 mM PTU for 24 h prior to injections (Karlsson et al., 2001).

### Infection assays

Infection assays were performed as previously described (Willis et al., 2018). For infection experiments, individual colonies were grown overnight in 5 ml TSB+100 µg/ml carbenicillin at 37°C, rotated at 200 rpm. To obtain bacteria grown to exponential phase, i.e. to OD_600_ (∼0.6), 400 µl of overnight culture was diluted in growth medium supplemented with 20 ml of TSB+100 µg/ml carbenicillin and grown for 2 h when culturing *S. flexneri* M90T, or for 90 min when culturing *S. sonnei* 53G. The bacterial subculture was then centrifuged at room temperature for 4 min at 4000 ***g***. The obtained bacterial pellet was resuspended in 1 ml 1× PBS, centrifuged for 1 min at 6000 ***g*** and the pellet resuspended in 400 µl PBS. OD_600_ was measured using a PBS-diluted (×50) sample of this bacterial suspension.

The OD_600_ of the suspension was then adjusted in 50/50 volumes of 0.5% Phenol Red/4% polyvinylpyrrolidone (PVP) to, respectively, reach OD_5_ or OD_40_, i.e. 2500 colony-forming units (CFUs) or >10,000 CFUs.

Before injection, larvae were prepared at 2 dpf and, if necessary, dechorionated. They were anesthetised in 0.4 mg/ml (2×) tricaine. Of the suspension to inject – i.e. PBS control or bacterial suspension – 5 µl was loaded into a glass capillary needle that was manually opened to inject 1 or 2 nl into the larvae hindbrain ventricle (HBV). Injection was performed for 200 milliseconds, by using nitrogen (N_2_) at pressure between 35 and 40 psi.

### Morpholino oligonucleotide injection

Modified antisense morpholino (MO) oligonucleotides were used to deplete leukocyte populations in the transgenic zebrafish line Tg(*lyz*::DsRed)*^nz50^* by knocking down expression of the *pu.1* transcription factor (Tenor et al., 2015). An MO oligonucleotide without any known target in the zebrafish genome was used as control (Mostowy et al., 2013). Solutions were diluted to the desired concentration of 1 mM in 0.1% Phenol Red solution (Sigma-Aldrich) and 1–5 nl (1–5 pmol) were microinjected into the yolk sack between cell stages 1 and 2.

### Quantification of bacterial burden

To quantify and characterize the bacterial inoculate, three injected larvae per condition were individually transferred into microcentrifuge tubes containing 200 µl Triton X-100 (0.1%) diluted in PBS. The larvae were then mechanically disrupted and dissolved in the solution by using sterile pestles. The suspensions were serially diluted by 10 to reach a final dilution of 10^5^, and 20 µl were plated on TSA plates supplemented with 100 µg/ml carbenicillin and 0.01% Congo Red, followed by incubation for 18 h at 37°C. CFUs were hand counted the next day to assess the bacterial burden in infected larvae.

### MICs determination and antibiotic treatment assays

To assess the *in vitro* minimum inhibitory concentrations (MICs) for every antibiotic tested on the two *Shigella* strains, i.e. *S. flexneri* M90T and *S. sonnei* 53G, antibiotic stock solutions were prepared, containing 8 µg/ml nalidixic acid (NAL), 160 µg/ml chloramphenicol (CM), 16 µg/ml azithromycin (AZI) or 2 µg/ml trimethoprim (TPI). These stock solutions were then five times serially diluted 1:1 with TSB, to generate five individual daughter solutions comprising the following concentration of antibiotic. NAL: 4, 2, 1, 0.5, 0.25 and 0.125 µg/ml; CM: 80, 40, 20, 10, 5 and 2.5 µg/ml; TPI: 1, 0.5, 0.25, 0.125, 0.063 and 0.032 µg.ml; AZI: 8, 4, 2, 1, 0.5 and 0.25 µg/ml. Of every solution 200 µl was transferred into the wells of several 96-well plates. Per well, 1 µl of overnight bacterial culture was then inoculated under every condition. Control wells contained 200 µl of TSB only. For every condition, triplicate set-ups were tested, with 96-well plates sealed and incubated at 37°C for 7 h under rotation at 200 rpm. Thereafter, bacterial growth for each condition was assessed visually. The MICs of antibiotic were considered to be those present within the first well without noticeable bacterial growth. Importantly, only the MICs of antibiotics against *S. sonnei* 53G, i.e. the highest concentration of each antibiotic, were used for antibiotic treatment assays *in vivo*.

To test the effect of antibiotics at these concentrations, ten larvae were grown in a 6-well plate for 24 h, in E2 medium supplemented with the corresponding antibiotic at the *S. sonnei in vitro* MIC. Developmental defects were evaluated by observation under a stereomicroscope.

For *in vivo* assays of antibiotic treatment, larvae injected with 10,000 CFUs of *Shigella* were kept for 30–60 min in antibiotic diluted in 0.5× E2 medium supplemented with 0.2 mg/ml tricaine (1×) to reach the *in vitro* MIC of the respective antibiotic. This first antibiotic treatment was performed while preparing the remaining experimental setup. When ready, larvae were placed in 96-well plates (CellCarrier-96 Ultra; PerkinElmer) and embedded by hand in 1% low melting-point agarose (diluted in 0.5× E2 medium) for immobilisation. For whole-fish imaging, larvae were placed horizontally and on their lateral side; for HBV imaging larvae were placed with the HBV against the glass bottom of the plate. Once the agarose was set, wells were topped up with respective antibiotic solution diluted in E2+0.2 mg/ml tricaine (1×) at twice the concentration of the *in vitro* MIC for the appropriate antibiotic. Since agarose interferes with the diffusion of the antibiotic, this double dose of antibiotic is necessary to obtain an effect identical to that observed for free-swimming larvae.

### Microscopy imaging

Larvae were imaged *in vivo* in a 96-well plate, using a Zeiss Celldiscoverer 7 (CD7) microscope. For imaging, larvae were individually positioned in wells with a drop of 0.5× E2 medium containing 1× tricaine without or supplemented with antibiotics and were then embedded in 1% low melting agarose. For whole-zebrafish imaging, agarose was added to an entire row (12 wells) and larva positions were manipulated, so that larvae lay on their side and horizontally in the well. For HBV imaging, agarose was added to 3–6 wells at the same time and each larva was turned upside down with their HBV against the bottom of the plate. For time-lapse experiments, images were taken every 2 h until 24 h post-infection (hpi), with the CD7 platform heated to 31°C. Whole-zebrafish imaging was performed using a 5×/0.35 plan-apochromat objective with a 0.5× tube lens to acquire a single *z*-plane image. Imaging of HBVs was performed with the same objective but by using a 2× tube lens to capture 51 slice *z*-stacks over 250 µm. Bacterial fluorescence and neutrophils of the HBV were captured simultaneously. To compare the performance of the ‘Ellett and Lieschke’ method in images acquired with the CD7 microscope or a Leica M205FA stereomicroscope, larvae were imaged using a Leica M205FA stereomicroscope at ×0.3 magnification.

### Image analysis

Custom macros were designed in Fiji (https://fiji.sc) to automate image analysis. All macros are dependent on information (including folder containing the files to analyse, channel to analyse, parameters to consider, type of analysis desired) provided by the user via a user-friendly interface.

#### Quantification of bacterial burden

The macro used to quantify the bacterial burden was designed to analyse *z*-stacks of larval HBVs; however, it can be adapted to analyse many other types of image, such as whole-zebrafish images. The macro automatically performs a sum slices *z*-projection on the images, measuring the mean fluorescence within the entire *z*-stack as well as the image area, then multiplying both values to obtain the total fluorescence within the *z*-stack. The macro automatically runs through files in batches and returns a text document listing the title of all images analysed and the total fluorescence associated to each. This total fluorescence value is considered to be a proxy of the bacterial burden. As this macro also measures the background fluorescence, it is important to look only at normalised – not raw – values, providing the advancement of fluorescence over time. Raw values are the sum of background fluorescence and HBV fluorescence and, thus, are not suitable indicators of the HBV fluorescence. However, as background fluorescence does not change over time, these values can be used to represent the advancement of fluorescence in the HBV over time.

#### Neutrophil detection and characterisation (LenCell)

Neutrophil detection and characterisation can be assessed by two different macros, depending on the image type to analyse, single-plane or *z*-stack image. We used single-plane images to count neutrophils at whole-animal level, whereas neutrophils in HBVs were counted using *z*-stack images. In brief, the macro for single-plane images automatically performs a bandpass filter with the ‘subtract background’ feature of Fiji. This feature uses the ‘Rolling Ball Background Subtraction’, which iteratively determines local background values for every pixel in the image, by averaging the values over a large ball around the pixel. This local background value is then subtracted from the image. The radius of the ‘rolling ball algorithm’ (RBA) can be changed directly in Fiji and in the macro. To apply the bandpass filter, the macro duplicates the original image and subtracts the backgrounds of each of these duplicates using different RBA values. Typically, a large RBA value is chosen (in this work, an RBA of 11 was used) for one duplicate, to remove large background spots (e.g. yolk sack, skin, contaminations in the agarose), and a small RBA value (in this work, an RBA of 3 was used) is chosen for the other duplicate, to remove the ‘small’ background, especially at neutrophil clusters. Finally, to retrieve a cleaned result image that is similar to the original one, the pixel values of both duplicates are multiplied. The image obtained after this operation is automatically converted to a binary mask, using a default threshold. This threshold can be modified for other applications. The binary mask is extensively cleaned with a step of opening (this is a Fiji function that consists of an erosion followed by a dilation of the particles detected in the binary image, i.e. the black spots), which removes noise. The resulting image is then processed through a step of watershed segmentation that automatically detects clusters of black spots and divides them.

The cleaned binary image is finally processed by the ‘analyse particle’ tool of Fiji, which automatically detects objects in the binary mask above the threshold or regions of interest (ROIs) in the image, and stores information about their coordinates, shape and size. A size filter set is applied (in this work, the filter size was from 0 to ∞), and the number of ROIs is automatically counted, while their boundaries are overlaid on the original image. This provides a visual output to check for any artifact. Additionally, a compressed file containing the positional information of the ROIs detected is automatically saved on the computer.

These steps are entirely automated, although the user must provide the value of the wanted parameters, i.e. the two RBAs to use and the minimum size of the size filter, as well as the path to the folder containing the files to analyse. After processing, a table containing the titles of the analysed images and the number of ROIs associated is returned, as well as a series of visual outputs.

This macro can additionally be automatically optimised. To do so, the user hand counts a series of images and provides data to the macro. After asking the range of parameters, i.e. RBA values and size filter, the user wants to train the macro on, it will automatically and iteratively use every combination of parameters to count neutrophils in the provided images. The parameters that provide results closest to those obtained by hand counting are then considered as ‘optimized parameters’.

The macro to count and detect neutrophils in an HBV *z*-stack image is very similar; however, it includes several pre-processing steps. First, the image is cleaned by using a very large band pass filter (first RBA: 100, second RBA: 200). The two resulting images are added up, and the resulting image is processed through a *z*-projection by standard deviation, which makes the ROIs stand out. Finally, the *z*-projection is processed through the same pipeline as described before for whole-zebrafish images but with slightly differing parameters (first RBA: 9, second RBA: 10, minimum size 0).

Although a correction factor can be calculated to limit the error rate and determine absolute neutrophil numbers, we avoid post-processing correction of the data and only present the raw, uncorrected results (leukocyte units) throughout this manuscript for both the LenCell macro and ‘Ellett and Lieschke’ method.

Dissemination, i.e. bacterial spreading out of the HBV (Fig. 4), was assessed manually. A dissemination event is defined as the presence of green fluorescence in the spinal cord of larvae infected with GFP-expressing *Shigella*.

### Statistical analysis and data processing

Statistical significance was determined using GraphPad Prism v9. For comparisons of the different analysis methods, a two-way ANOVA with post-hoc Sidak's multiple comparisons test was performed. For experiments occurring over time, i.e. bacterial burden and neutrophil counts over time, values are presented as normalised to the first time point for every larva (4 hpi). Two-way ANOVA with post-hoc Dunnett's multiple comparisons test was performed for these experiments. For quantification of spreading events, unpaired two-tailed Student's *t*-test was used. Outlier larvae (dead, uninfected) were removed from the dataset. The last timepoint typically contains more dead larvae and, therefore, fewer replicates. In figures, *P*-values are as follows: *P*>0.05, non-significant (ns); *P<0.05, **P<0.01, ***P<0.001, ****P<0.0001.

## Supplementary Material

10.1242/dmm.049908_sup1 Supplementary information
