# Supplementary material for: An automated microscopy workflow to study Shigella–neutrophil interactions and antibiotic efficacy in vivo
Source: Dis Model Mech. 2023 May 10;16(6):dmm049908. doi: 10.1242/dmm.049908 (PMC10184671; doi:10.1242/dmm.049908)
Supplement: Supplementary information [file dmm-16-049908-s1.pdf]

Figure S1

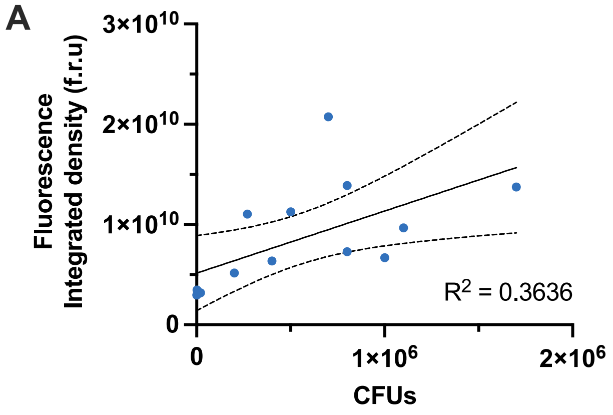

Fig. S1. Quantification of bacterial burden in the HBV of PTU-treated larvae. Data collected in the HBVs of *S. flexneri* M90T-infected 2 dpf zebrafish larvae previously treated with PTU. Total fluorescence in the Z-stack image of the infected zebrafish larvae HBVs, at different infectious doses, measured at 2 and 24 hpi, and correlated to the precise number of CFUs (experimentally assessed), n = 14. Full dark line: linear regression. Dashed lines: 95%confidence intervals.

Figure S2

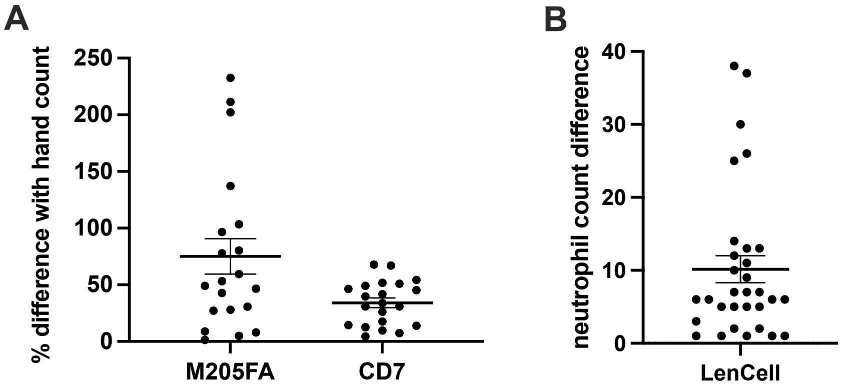

Fig. S2. Efficiency of the Ellett and Lieschke method using different imaging set-ups and of the Lencell method to count leukocyte units in the HBV. (A) Images collected using Leica M205FA stereomicroscope or Zeiss CD7 to image M90T injected 2 dpf larvae at 24 hpi. The Ellett and Lieschke method is used here to determine leukocyte units in the larvae. The error is defined by the absolute value of the percentage of the difference between hand-counted neutrophils and the count provided by this method. Black bars: mean ± SEM. (B) LenCell was used to determine neutrophils in the HBV of zebrafish larvae injected with *S. flexneri* GFP. Absolute differences between hand counts and LenCell counts are plotted. n=30 images (51 slices z-stack), black bars: mean ± SEM.

Figure S3

A

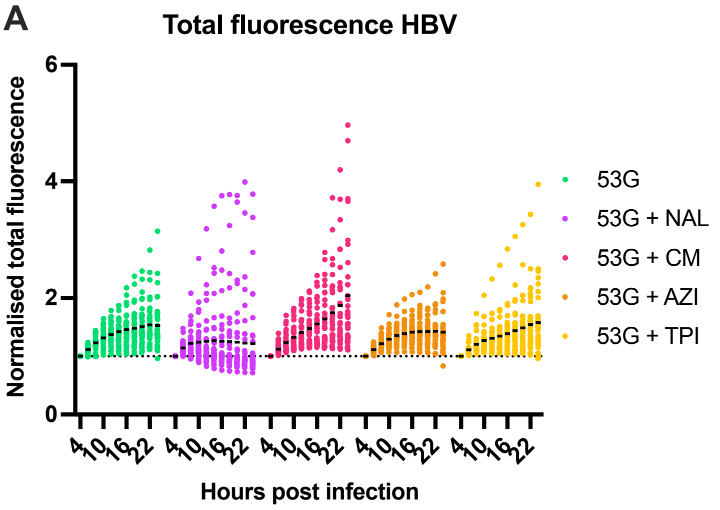

	Hours post infection									
	**6**	**8**	**10**	**12**	**14**	**16**	**18**	**20**	**22**	**24**
**53G vs. 53G+NAL**	ns	ns	ns	ns	ns	ns	ns	ns	ns	ns
**53G vs. 53G+CM**	ns	ns	ns	ns	ns	ns	ns	ns	ns	*
**53G vs. 53G+AZI**	ns	ns	ns	ns	ns	ns	ns	ns	ns	ns
**53G vs. 53G+TPI**	ns	ns	ns	ns	ns	ns	ns	ns	ns	ns

B

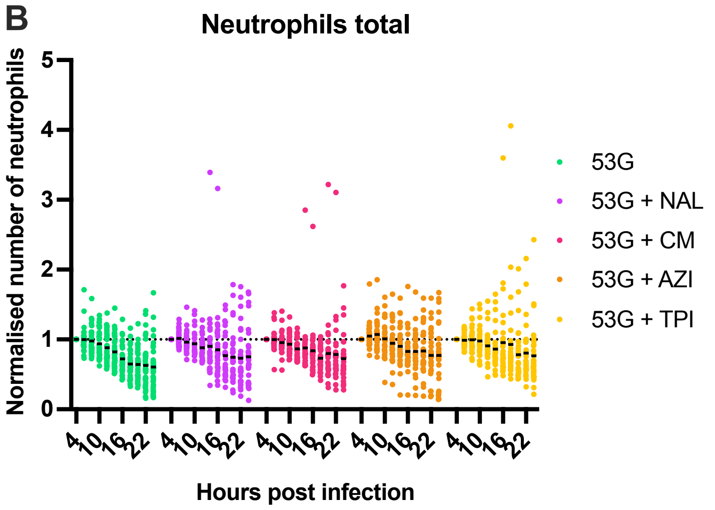

	Hours post infection									
	**6**	**8**	**10**	**12**	**14**	**16**	**18**	**20**	**22**	**24**
**53G vs. 53G+NAL**	ns	ns	ns	ns	ns	ns	ns	ns	ns	ns
**53G vs. 53G+CM**	ns	ns	ns	ns	ns	ns	ns	ns	ns	ns
**53G vs. 53G+AZI**	ns	ns	ns	ns	ns	ns	*	*	ns	ns
**53G vs. 53G+TPI**	ns	ns	ns	ns	ns	ns	ns	ns	ns	ns

C

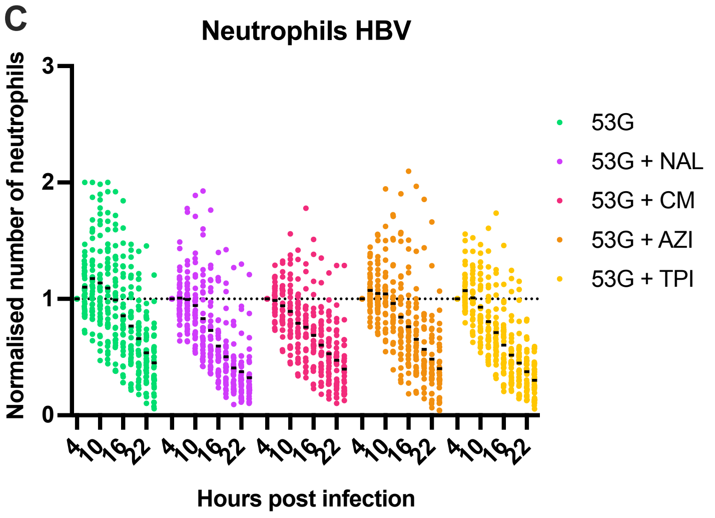

	Hours post infection									
	**6**	**8**	**10**	**12**	**14**	**16**	**18**	**20**	**22**	**24**
**53G vs. 53G+NAL**	ns	*	ns	*	*	**	**	**	*	ns
**53G vs. 53G+CM**	ns	**	*	**	*	ns	ns	ns	ns	ns
**53G vs. 53G+AZI**	ns	ns	ns	ns	ns	ns	ns	ns	ns	ns
**53G vs. 53G+TPI**	ns	*	*	**	**	**	**	*	*	*

Disease Models & Mechanisms • Supplementary information

Fig. S3. The impact of antibiotics on *Shigella sonnei* infection and on neutrophils. All data presented here is collected from *S. sonnei* 53G- infected 2 dpf zebrafish larvae, non-treated (green) and treated with Nalidixic Acid (NAL, purple), Chloramphenicol (CM, pink), Azithromycin (AZI, orange), or Trimethoprim (TPI, yellow). (A) Normalised total bacterial fluorescence in the HBV. Data normalized to the first timepoint (4 hpi). Data pooled from 3 independent experiments using n > 10 larvae per condition per experiment (ns: non-significant, *p < 0.05. Two-way ANOVA with Dunnett's multiple comparisons test). (B) Normalised neutrophil quantification at the whole larvae level. Data normalized to the first timepoint (4 hpi). Data pooled from 3 independent experiments using n > 10 larvae per condition per experiment (ns: non-significant, *p < 0.05. Two-way ANOVA with Dunnett's multiple comparisons test). (C) Normalised neutrophil quantification in the HBV. Data normalized to the first timepoint (4 hpi). Data pooled from 3 independent experiments using n > 10 larvae per condition per experiment (ns: non-significant, *p < 0.05, **p < 0.01. Two-way ANOVA with Dunnett's multiple comparisons test).

Figure S4

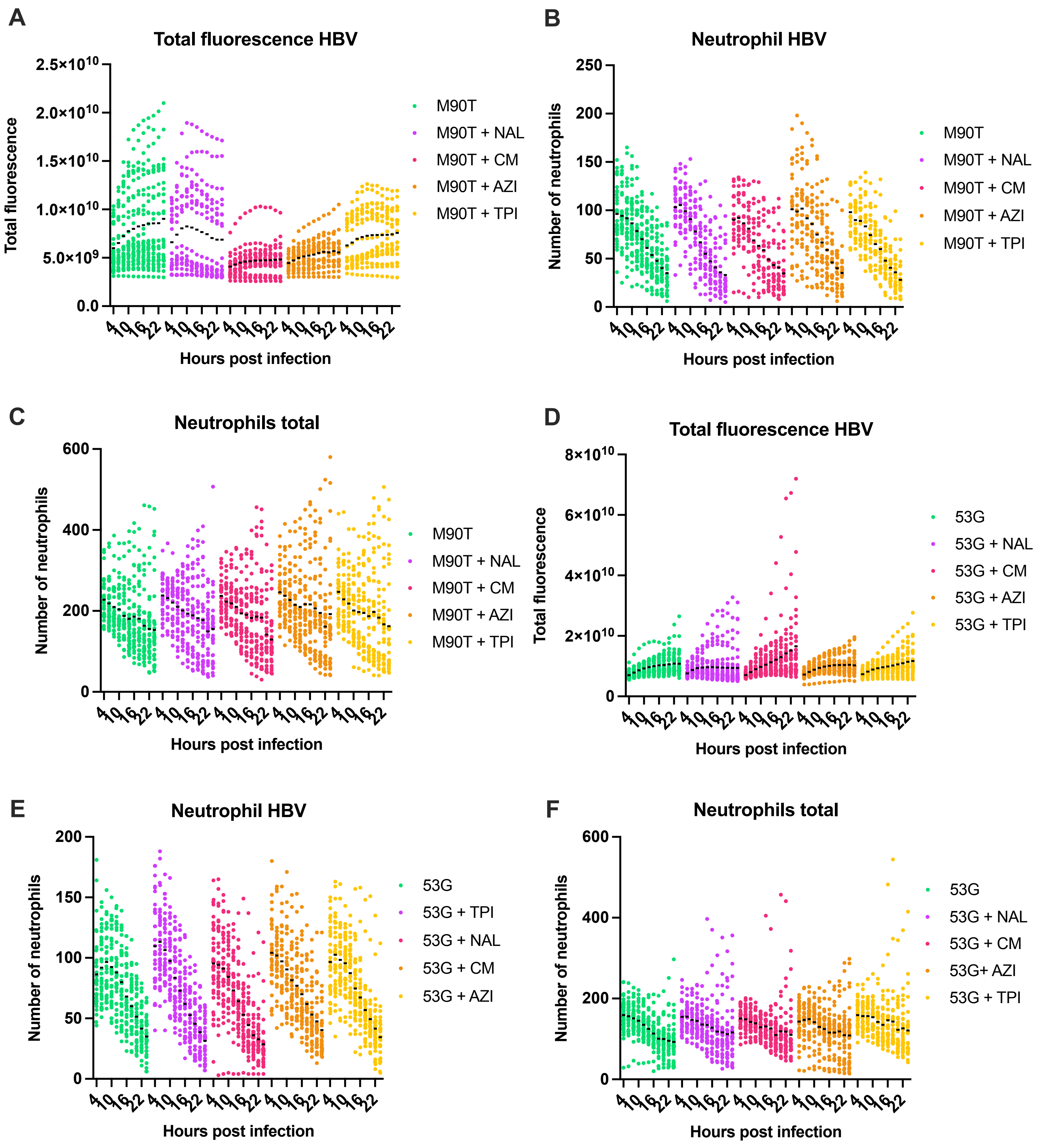

Disease Models & Mechanisms • Supplementary information

Fig. S4. Raw fluorescence reads of bacterial burden and neutrophil quantifications. All data presented here is collected from *Shigella* infected 2 dpf zebrafish larvae, non-treated (green) and treated with Nalidixic Acid (NAL, purple), Chloramphenicol (CM, pink), Azithromycin (AZI, orange), or Trimethoprim (TPI, yellow). (A) Total bacterial fluorescence of *S. flexneri* M90T in the HBV. Data pooled from 3 independent experiments using n > 10 larvae per condition per experiment (B) Neutrophil quantification at the whole larvae level during *S. flexneri* M90T infection. Data pooled from 3 independent experiments using n > 10 larvae per condition per experiment (C) Neutrophil quantification in the HBV of *S. flexneri* M90T infected larvae. Data pooled from 3 independent experiments using n > 10 larvae per condition per experiment. (D) Total bacterial fluorescence of *S. sonnei* 53G in the HBV. Data pooled from 3 independent experiments using n > 10 larvae per condition per experiment (E) Neutrophil quantification at the whole larvae level during *S. sonnei* 53G infection. Data pooled from 3 independent experiments using n > 10 larvae per condition per experiment (F) Neutrophil quantification in the HBV of *S. sonnei* 53G infected larvae. Data pooled from 3 independent experiments using n > 10 larvae per condition per experiment.

Figure S5

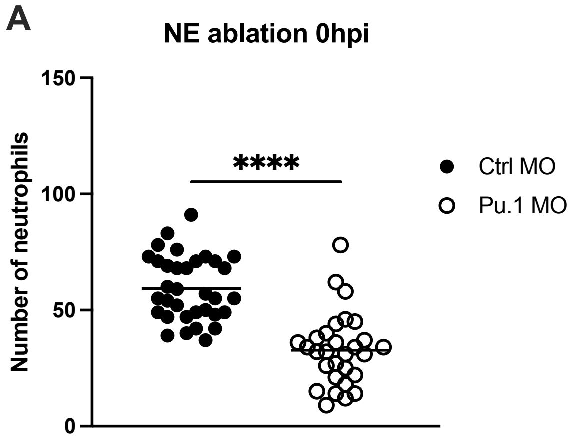

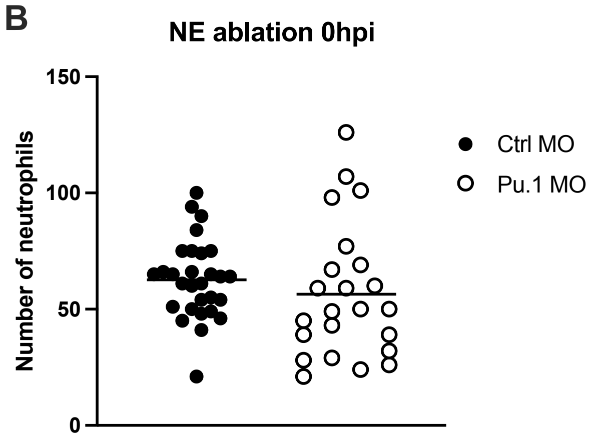

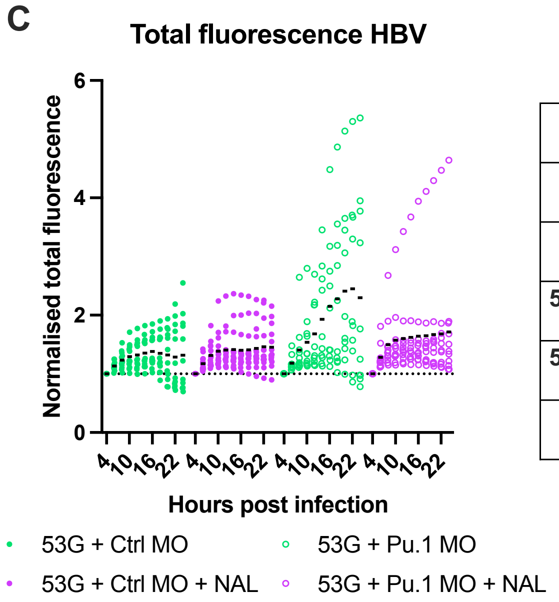

	Hours post infection									
	6	8	10	12	14	16	18	20	22	24
53G + Ctrl MO vs. 53G + Ctrl MO + NAL	ns	ns	ns	ns	ns	ns	ns	ns	ns	ns
53G + Ctrl MO vs. 53G + Pu.1 MO	ns	ns	ns	ns	ns	ns	ns	ns	ns	ns
53G + Ctrl MO vs. 53G + Pu.1 MO + NAL	ns	ns	ns	ns	ns	ns	ns	ns	ns	ns
53G + Ctrl MO + NAL vs. 53G + Pu.1 MO	ns	ns	ns	ns	ns	ns	ns	ns	ns	ns
53G + Ctrl MO + NAL vs. 53G + Pu.1 MO + NAL	ns	ns	ns	ns	ns	ns	ns	ns	ns	ns
53G + Pu.1 MO vs. 53G + Pu.1 MO + NAL	ns	ns	ns	ns	ns	ns	ns	ns	ns	ns

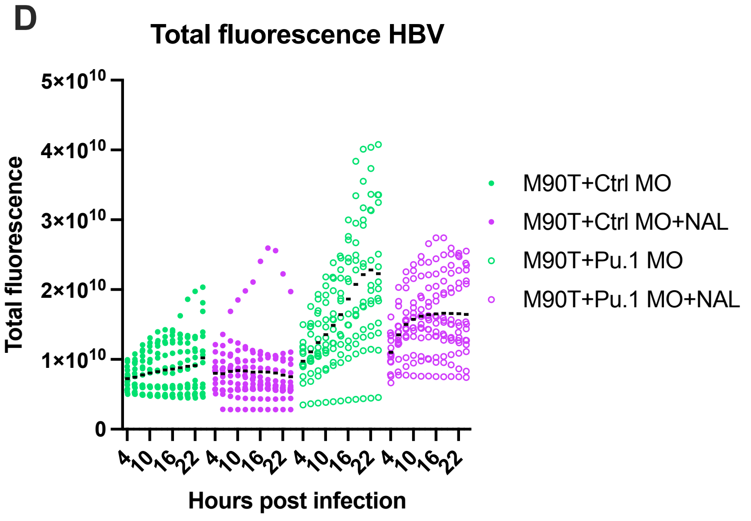

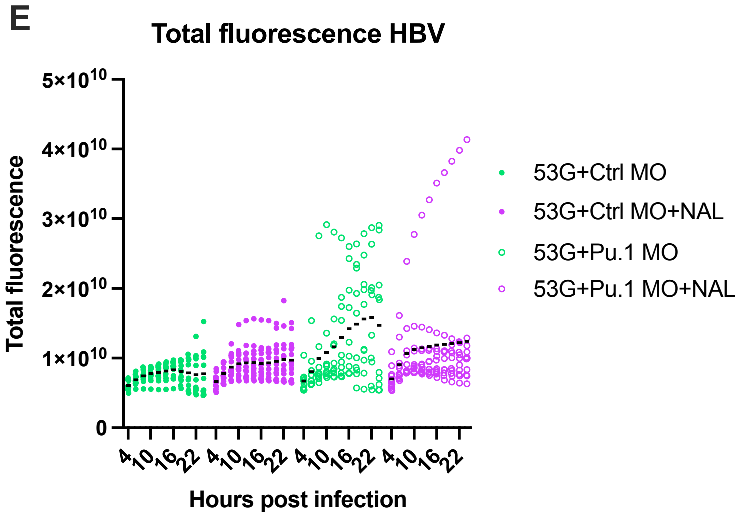

Disease Models & Mechanisms • Supplementary information

Fig. S5. Nalidixic acid and leukocytes work in an additive manner to control *Shigella* infection. (A) and (B) Efficiency of the ablation upon control (Ctrl MO, black full circles) and *pu.1* morpholino (Pu.1 MO, open circles) injections prior to *S. flexneri* M90T (A) and *S. sonnei* 53G (B) infection. Data pooled from 3 independent experiments using n > 3 larvae per condition (****$p < 0.0001$. Unpaired Student's t- test). (C) Normalised total *S. sonnei* 53G fluorescence in the HBV. Data presented collected from control (Ctrl MO, full circles) and *pu.1* morpholino (Pu.1 MO, open circles) injected larvae. *S. sonnei* 53G-infected 2 dpf zebrafish larvae were non-treated (green) and treated with Nalidixic Acid (NAL, purple). Data normalized to the first timepoint (4 hpi). Data pooled from 3 independent experiments using n > 3 larvae per condition per experiment (ns: non-significant. Two-way ANOVA with Tukey's multiple comparisons test). (D) and (E) Raw fluorescence reads of bacterial burden in *S. flexneri* M90T (D) and *S. sonnei* 53G (E) infected larvae. Data pooled from 3 independent experiments using n > 3 larvae per condition per experiment.

Disease Models & Mechanisms • Supplementary information

Table S1. Minimum inhibitory concentrations of antibiotics for S. flexneri and S. sonnei *in vitro*. Minimum inhibitory concentrations (in µg/ml) of Nalidixic Acid, Chloramphenicol, Azithromycin and Trimethoprim on *S. flexneri* and *S. sonnei*. These concentrations were determined *in vitro*.

	S. flexneri	***S. sonnei***
**Nalidixic Acid**	2.00	4.00
**Chloramphenicol**	0.62	2.50
**Azithromycin**	2.00	8.00
**Trimethoprim**	0.125	2.00

Disease Models & Mechanisms • Supplementary information
